# Comparative transcriptome analysis unveils unique antiviral immune signatures of *Rhinolophus pusillus*

**DOI:** 10.3389/fimmu.2026.1797102

**Published:** 2026-05-20

**Authors:** Lamei Zhao, Yelin Han, Yuyang Wang, Wenliang Zhao, Panpan Xu, Xiujuan Yu, Qing Tang, Jianing Ma, Kun Zhao, Shixuan Dong, Qi Jin, Zhiqiang Wu

**Affiliations:** 1 National Health Commission (NHC) Key Laboratory of Systems Biology of Pathogens, State Key Laboratory of Respiratory Health and Multimorbidity, National Institute of Pathogen Biology, Chinese Academy of Medical Sciences & Peking Union Medical College, Beijing, China; 2 College of Animal Science and Veterinary Medicine, Shenyang Agricultural University, Shenyang, China; 3 Key Laboratory of Pathogen Infection Prevention and Control (Ministry of Education), National Institute of Pathogen Biology, Chinese Academy of Medical Sciences & Peking Union Medical College, Beijing, China; 4 School of Population Medicine and Public Health, Chinese Academy of Medical Sciences & Peking Union Medical College, Beijing, China

**Keywords:** antiviral defense, *Rhinolophus pusillus*, sarbecovirus, viral tolerance, zoonotic disease

## Abstract

**Background:**

*Rhinolophus pusillus* is currently the only known horseshoe bat that simultaneously harbors SARS-CoV-related coronaviruses (SARSr-CoVs), SARS-CoV-2-related coronaviruses (SC2r-CoVs), and a sarbecovirus recombinant lineage with a mosaic genome combining features of both SARS-CoV and SARS-CoV-2. However, the molecular basis underlying multi-virus coexistence and viral tolerance in this species remains incompletely defined. Here, we focus on basal gene expression patterns to investigate the immune landscape of this species.

**Methods:**

Multi-tissue transcriptomes were generated from six individuals of *R. pusillus* and *Rhinolophus ferrumequinum*, and integrated with publicly available RNA-seq datasets from multiple mammalian species relevant to zoonotic disease. Cross-species comparative transcriptomic analyses were performed to assess basal immune activity. Weighted gene co-expression network analysis (WGCNA) was applied to identify tissue-specific regulatory modules, and differential expression analyses were conducted to characterize immune-related transcriptional differences. In addition, long non-coding RNA (lncRNA)-mRNA regulatory networks were constructed to explore coordinated immune regulation.

**Results:**

*R. pusillus* exhibited elevated basal transcriptional activity of innate antiviral immune processes compared with other mammals, with significant enrichment of genes involved in innate immune responses and antiviral defense. In contrast, transcriptional programs associated with inflammatory regulation were relatively attenuated. WGCNA identified distinct tissue-specific functional modules with coordinated regulatory architectures. *R. pusillus* displayed transcriptional features consistent with an enhanced capacity for viral infection tolerance relative to *R. ferrumequinum*. LncRNA analyses indicated coordinated regulation of inflammatory responses and DNA damage repair through lncRNA-mRNA regulatory networks.

**Conclusions:**

By integrating comprehensive transcriptomic analyses of protein-coding RNAs with the prediction of lncRNA, this study provides a systematic molecular framework based on constitutive gene expression for understanding the mechanisms of viral tolerance in *R. pusillus* under conditions of multi-virus coexistence. These findings advance our understanding of bat immunobiology and offer insights into infection-related immune adaptations in *R. pusillus* as a key viral reservoir species.

## Introduction

As the only mammals capable of powered flight, bats (*Chiroptera*) account for approximately 20% of all living mammal species, with more than 1500 species currently identified (https://www.batcon.org/). Bats exhibit extensive diversity in morphology, geographical distribution, ecological niches, diet, and social interactions, accompanied by a series of extraordinary adaptations (1), including laryngeal echolocation for navigation and prey detection, vocal learning, a low rate of tumorigenesis (2), exceptional longevity relative to body size (3), and distinct immune characteristics. While serving as natural reservoir hosts for over 100 viruses (4), bats generally remain asymptomatic and survive these infections (5). Their unique immune systems likely enable them to tolerate viruses that are lethal to other mammals, including henipaviruses (Nipah and Hendra viruses), coronaviruses (sarbecoviruses and merbecoviruses), and filoviruses (Ebola and Marburg viruses) (6). Such viral tolerance suggests the presence of regulatory mechanisms that balance antiviral responses with controlled inflammation (7–9), providing insights into host-pathogen coevolution and viral spillover potential.

Since the 21st century, the successive outbreaks of SARS, caused by SARS-CoV (10), and Coronavirus Disease 2019 (COVID-19), caused by SARS-CoV-2 (11), have underscored the ongoing global public health threat posed by sarbecoviruses, a subgenus of *Betacoronavirus* for which horseshoe bats (family Rhinolophidae) serve as the main natural reservoirs (12–14). Despite ongoing surveillance efforts, effective strategies to mitigate the spillover risk of these viruses remain highly limited. *R. pusillus* is currently the only known horseshoe bat species that concurrently harbors SARSr-CoVs, SC2r-CoVs, and recombinant strains (termed the L-R lineage) (15). Notably, this distinctive viral repertoire contrasts with that of other closely related horseshoe bat species, in which sarbecovirus diversity appears more restricted. This unique pattern of viral coexistence provides an opportunity to investigate mechanisms that support long-term viral persistence. This may suggest that the potential risks of zoonotic virus spillover might occur at the horseshoe bat-human interfaces, increasing the possibility of outbreaks of emerging infectious diseases.

*R. pusillus* represents the smallest-bodied species within the genus *Rhinolophus*, characterized by a wide geographic distribution, relatively large population sizes, pronounced sociality, and substantial genetic differentiation across geographic regions. Despite increasing attention to its exceptional capacity to harbor multiple viruses concurrently, fundamental biological knowledge of *R. pusillus* remains limited. In particular, the paucity of comprehensive genomic resources and multi-tissue transcriptomic datasets has impeded a holistic understanding of *R. pusillus*, constraining investigations into its evolutionary dynamics, ecological adaptations, host-virus interactions, and immune regulatory mechanisms. Accordingly, these limitations highlight an urgent need for integrative omics-based investigations to systematically explore the molecular mechanisms underlying the coexistence of diverse sarbecoviruses in *R. pusillus*.

In this study, we generated multi-tissue transcriptomes of *R. pusillus* and conducted comparative transcriptomic analyzes across mammalian species to characterize its basal transcriptomic landscape. We examined global transcriptional features associated with antiviral immunity and identified molecular signatures linked to viral tolerance and sustained viral coexistence in this species. By filling a critical knowledge gap for this ecologically significant bat, our study provides a valuable genetic resource and molecular framework for elucidating the mechanisms of viral tolerance and the evolutionary dynamics of sarbecoviruses within their natural hosts.

## Materials and methods

### Collection of tissue samples and RNA preparation

A total of six adult male horseshoe bats, including three *R. pusillus* and three *R. ferrumequinum*, were wild-caught from four provinces in China (Yunnan, Guangxi, Beijing, and Zhejiang) between 2020 and 2023. All bats were captured in the summer and appeared healthy and active at the time of capture. Age was assessed based on body weight, morphology, and skeletal development, and all individuals were confirmed as healthy adults, within the typical infection age range for these species and suitable for basal immune analyzes due to their stable immune status. All bats were collected from natural cave roosts in suburban or rural mountainous regions, which represent typical ecological habitats for horseshoe bats. A focused virome assessment was performed on the lung and intestine tissues of the bats, in which virus-associated reads were screened and further analyzed (**Supplementary Figure 1**). Species identification was initially performed based on morphological characteristics and subsequently confirmed for each individual by molecular identification using the mitochondrial cytochrome b (cytb) gene. Following species determination, bats were anesthetized prior to humane euthanasia by cervical dislocation. Tissue samples from eight organs (heart, liver, intestine, spleen, kidney, lung, brain, and muscle) were rapidly collected, transferred into RNase-free PCR tubes containing preservation medium, and immediately stored at -80 °C until RNA extraction. Total RNA was extracted with the MagZolTM Reagent (Axygen, USA) following kit directions. RNA degradation and contamination were assessed by electrophoresis on 1% agarose gels. RNA purity was evaluated using a NanoDrop 2000 spectrophotometer (NanoDrop, Wilmington, DE, USA), and RNA integrity was assessed with an Agilent 2100 Bioanalyzer (Agilent Technologies, USA). The RNA concentration was quantified using the Qubit RNA Assay Kit on a Qubit 4.0 Fluorometer (Life Technologies, CA, USA).

### Library construction and Illumina sequencing

Each mRNA-Seq Illumina sequencing library construction met the criteria of having > 2µg of total RNA, with the concentration of RNA≥200 ng/µL. Ribosomal RNA (rRNA) was removed from total RNA using the Ribo-Zero Gold Kit (Illumina, USA) according to the manufacturer’s instructions, thereby enriching for mRNA and other non-rRNA transcripts. The rRNA-depleted RNA was fragmented into approximately 200 bp fragments using fragmentation buffer. First-strand cDNA synthesis was performed using random hexamer primers and reverse transcriptase, followed by second-strand synthesis to generate double-stranded cDNA. Subsequently, a total of 48 sequencing libraries were prepared using the VAHTS Universal V6 RNA-seq Library Prep Kit for Illumina (Vazyme, China) following the manufacturer’s recommendations. After quality assessment, libraries were paired-end sequenced on an Illumina NovaSeq6000 platform, which generated 150-bp long paired-end reads per sample.

### Transcript processing and annotation

The raw data (short reads) of transcriptional sequencing for the bat tissues were processed with FASTP (0.23.0) (16). Clean reads were obtained by removing reads containing adapter, reads containing poly-N (N≥10%), and low-quality reads using FASTP software with default parameters. The GC content and overall quality metrics of the resulting clean reads were subsequently assessed, and only high-quality clean reads were retained for downstream analyses. The index of the reference genome was built using the HISAT2-build software, and clean reads for each sample were aligned to their corresponding reference genomes using HISAT2(2.1.0) (17). Alignment quality and library characteristics were evaluated with RSeQC (v3.0.1) (18) according to mapped reads. Genome-guided transcript reconstruction was performed using StringTie (19). Functional annotation of predicted genes was conducted by Diamond BLASTX (20) searches against multiple public databases with an e-value<1e–5, including STRING, Swiss-Prot, Gene Ontology (GO), KEGG, and the NCBI non-redundant (Nr) protein database. To enable cross-species transcriptomic comparisons, a reciprocal best hit (RBH) method was used to identify human orthologs of other mammals, based on human protein sequences from the Ensembl database. Briefly, protein sequences from each non-human species were aligned against the human proteome using BLASTP, and the top hit with an E-value < 1e−5 was retained. Reciprocal BLASTP searches were then performed from human to each non-human species under the same criteria. Gene pairs were defined as orthologs only if they were reciprocal best hits in both directions. Only one-to-one orthologs were retained, and transcripts with confidently assigned human orthologs were used for subsequent comparative analyses.

### Construction of multi-species spleen expression matrices

In addition to the spleen samples collected from two horseshoe bat species in this study, spleen transcriptomic datasets from other mammalian species were obtained from public databases. Specifically, raw RNA-seq data of spleen tissues from seven zoonosis-related mammals, including *Pteropus alecto*, *Mus musculus*, *Oryctolagus cuniculus*, *Canis lupus familiaris*, *Felis catus*, *Sus scrofa*, and *Bos taurus*, were downloaded from the Gene Expression Omnibus (GEO) database (https://www.ncbi.nlm.nih.gov/gds). Gene expression matrices of spleen tissues from healthy human individuals (*Homo sapiens*) were obtained from the GTEx database (https://www.gtexportal.org/). After quality filtering with fastp, clean data were aligned to the reference genome using HISAT2, and the resulting alignments were processed with SAMtools (21). Transcript assembly and abundance estimation were performed for each sample using StringTie. Gene-level raw read counts were obtained using featureCounts (22). Only genes with ≥1 count-per-million (CPM) in at least two samples were kept. Transcript abundances were normalized with Transcripts Per Million (TPM) mapped reads, and the TPM matrix was transformed by log2(TPM + 1).

### Weighted gene co-expression network analysis

Co-expression network analysis of gene expression was performed using the WGCNA R package (v.1.69) (23). Briefly, an expression matrix was constructed from 24 tissues of *R. pusillus*. Weighted correlations between all gene pairs were calculated to assemble a scale-free network. A hierarchical clustering tree was constructed based on these correlations, with branches representing distinct gene modules. The soft-thresholding power (β) was determined based on scale-free topology criteria. Gene modules were detected using step-by-step and dynamic tree cutting methods, with a minimum module size of 30 and a mergeCutHeight of 0.25. Module eigengenes (MEs) were calculated for each module, and module membership was determined based on the correlation between individual genes and their module eigengenes. The module with the highest correlation to each tissue was defined as the tissue-specific module. Functional enrichment analyses of genes in tissue-specific modules were performed using Metascape (https://metascape.org/gp/) (24) to interpret the biological roles of each module.

### Analysis of differentially expressed lncRNAs and mRNA

To evaluate differential expression patterns between *R. pusillus* and other species (including *R. ferrumequinum*), one-to-one orthologous genes were first identified using a reciprocal best hit (RBH) approach based on protein sequence similarity, following a procedure similar to that described above for human ortholog identification. Briefly, protein sequences between *R. pusillus* and each species were aligned in both directions using BLASTP, and only reciprocal best hit pairs with an E-value < 1e−5 were retained. Only one-to-one orthologs were used for downstream analyses. DEGs were identified using DESeq2 (25). We followed the DESeq2 recommended pipeline to create a gene-level count matrix by importing the Salmon quantification data using tximport (26). Genes with 0 counts across all samples were filtered out. The screening criteria for differentially expressed mRNAs and lncRNAs in each comparison group were: |log2 fold change|≥1 and a false discovery rate (FDR)<0.05, these were considered as significantly differentially expressed transcripts.

### Identification of hub IRGs in *R.pusillus* spleen

GO enrichment analysis was performed to annotate genes that were significantly up-regulated or down-regulated in the spleen of *R. pusillus* compared with *R. ferrumequinum*. We focused on a shared gene set involved in innate immune-related antiviral responses, which constituted the primary biological processes of interest in this study. The pathway expression networks (circular plots), as well as differential expression genes heatmaps and bubble plots, were generated using R software packages. To further identify immune hub genes in *R. pusillus*, protein-protein interaction (PPI) analysis was conducted for GO terms associated with innate immune responses using Cytoscape (27). To increase the reliability of hub gene identification, CytoHubba (28) was employed to screen the significant genes based on different algorithms, and the intersection of their results was selected as candidate hub IRGs. An UpSet plot was subsequently employed to screen hub genes supported by six independent topological algorithms, resulting in the identification of 16 robust immune hub genes. In addition, the GeneMANIA database (https://genemania.org) was used to explore genes or proteins that potentially interact with or are co-expressed with these hub genes.

### Identification of immune−related lncRNAs

We used the following steps to identify lncRNAs from non-redundant GTF files. According to the assembled transcript, the criteria of the minimum length greater than or equal to 200 nucleotides and at least 2 exon count thresholds were applied to screen the lncRNA candidates. Then, candidate lncRNA transcripts were selected by comparing gene annotation information of the reference sequence produced using Cuffcompare software (29). Here, we predicted the protein coding ability of lncRNA transcripts through three methods: Codin-Non-coding Index (CNCI), CPAT (Coding Potential Assessment Tool), and PLEK (predictor of long non-coding RNAs and mRNAs based on k-mer scheme). Based on those mapping procedures, the lncRNA profiles of 1249 orthologous lncRNAs were determined. The list of IRGs was based on the ImmPort database (https://immport.niaid.nih.gov). LncRNAs and IRGs with low expression levels were filtered out. Next, a Pearson correlation analysis was conducted between IRGs and lncRNAs. During this analysis, only lncRNAs with |R| > 0.6 and P < 0.001 were identified as immune-related lncRNAs (irlncRNAs). Finally, 562 irlncRNAs were included in this investigation.

### Predicting the target genes of differentially expressed lncRNAs

For a more comprehensive understanding of the differentially expressed lncRNAs, their target genes were predicted. lncRNAs can regulate the expression levels of target protein-encoding genes in trans and in cis regulation. Trans-regulated targets can be predicted based on mRNA sequence complementarity and RNA duplexenergy assessments. First, BLAST analysis was per formed to select similar or complementary sequences, after which RNAplex (30) was used to calculate the complementary energy between both sequences, and sequences above the threshold were selected as candidate targets. To identify lncRNA cis-regulated target genes, we selected genes regulated by cis-regulation that were 10 kb upstream and downstream from the lncRNA as the target genes. To better understand the functions of differentially expressed lncRNAs, the putative target genes were then used for enrichment analysis.

## Result

### Summary statistics of sequencing dataset and functional annotation

In this study, we generated a large-scale gene expression profile covering eight tissue types from *R. pusillus* and *R. ferrumequinum* (**Supplementary Table 1**), which represent major organ systems (i.e., nervous system, circulatory system, digestive system, urinary system, respiratory system, muscular system, and immune system). The sample information and tissue types included in this study dataset are provided in **Supplementary Table 2** and **Supplementary Figure 2**. We did not detect transcriptomic evidence suggestive of overt active viral infection in any of the six bats. Consistent with field observations, all individuals appeared healthy and active (**Supplementary Table 3**, **S4**). This study generated approximately 4.5 billion raw paired-end reads, with an average of 94 million reads per sample. After quality control, a total of 558 GB of high-quality data was kept for subsequent analysis. Based on the reference genomes of *R. pusillus* and *R. ferrumequinum* (mRhiFer1_v1), the high-quality sequencing reads were mapped using the HISAT2 software with the default setting. On average, 82.6% and 85.83% of clean reads were successfully mapped to the *R. pusillus* and *R. ferrumequinum* genomes, respectively, of which 76.87% and 82.87% were uniquely aligned (**Supplementary Table 5**). Only reads that were uniquely and concordantly mapped were retained for downstream analyses. Functional annotation of *R. pusillus* transcripts revealed that a total of 19,045 genes were annotated using the NR database, while 15,902 and 18,656 genes were annotated in Pfam and Swiss-Prot databases, indicating a high coverage of functional annotation across multiple databases. To facilitate subsequent comparative transcriptomic analyses, an all-against-all BLAST reciprocal best hits (RBH) strategy was applied, based on the human genome annotation, to identify orthologous genes between protein-coding genes annotated in the bat reference genomes and human protein sequences obtained from the Ensembl database. The resulting human-bat orthologs were subsequently used for downstream multi-species comparative transcriptomic analyses.

### Cross-species comparative transcriptomic analysis

**Figure 1:**
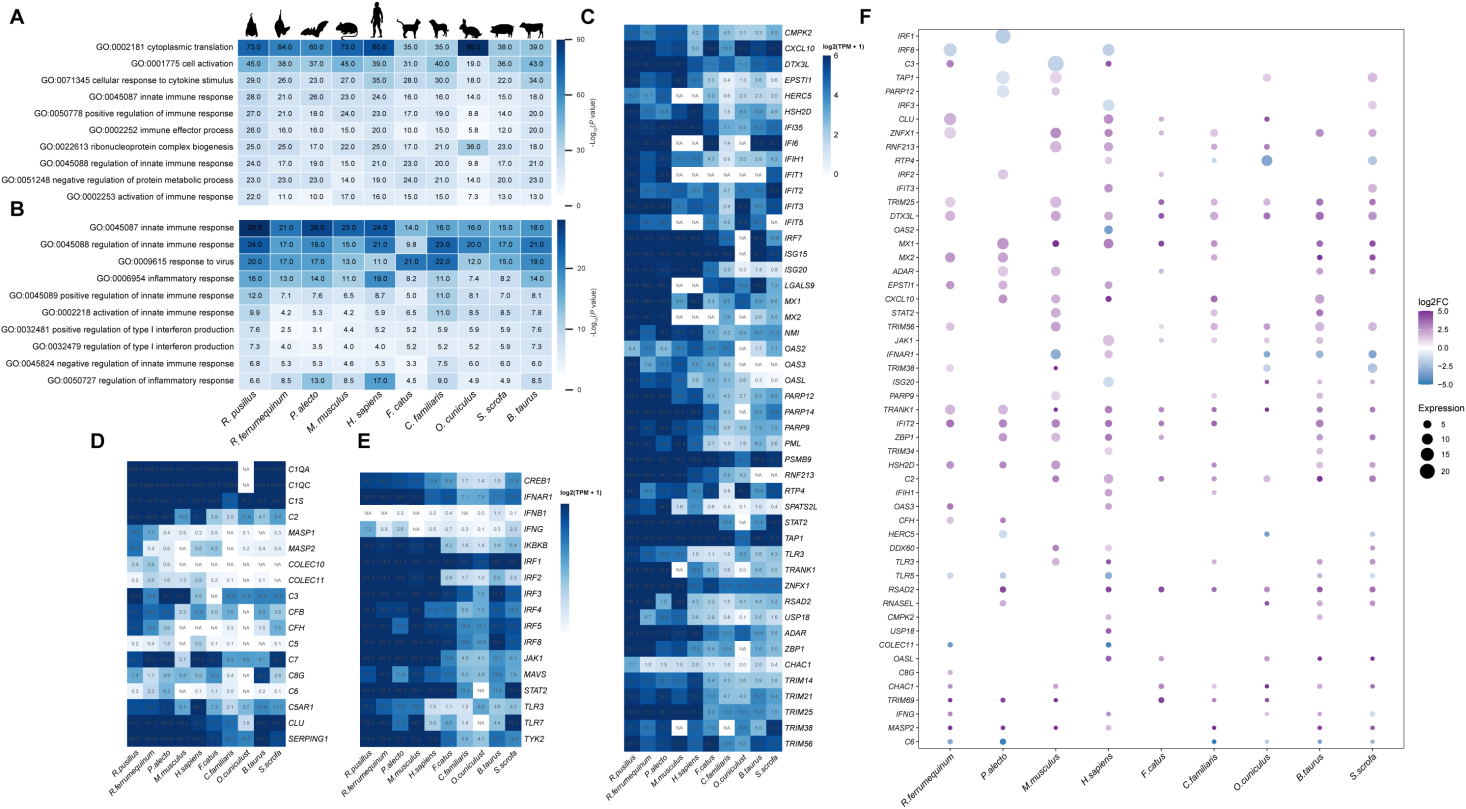
Comparative transcriptomic analysis of spleen tissues across multiple mammalian species. **(A)** Gene Ontology **(GO)** enrichment analysis of the top 1,000 highly expressed splenic genes from each of the ten mammalian species. The top ten significantly enriched GO terms are shown for each species. Color intensity represents the degree of enrichment, expressed as -log_10_ (P-value), and the specific enrichment value for each term is shown in the heatmap. **(B)** comparative enrichment patterns of antiviral innate immune-related GO terms across species, Numerical values displayed in the figure represent enrichment levels for each GO term in the corresponding species. **(C)** Heatmap of relative TPM for interferon-stimulated genes (ISGs). **(D)** Heatmap of relative TPM for complement system components. **(E)** Heatmap of relative TPM for components of the IFN signaling pathway. **(F)** Bubble plot of differential expression of antiviral immune genes between *R. pusillus* and nine mammalian species. Bubble size represents expression level (TPM). Negative log2FC values are shown in blue, and positive values are shown in purple. For all pairwise comparisons, each indicated mammalian species was used as the control group, and *R. pusillus* was used as the testing group. Positive log2FC values indicate higher expression in *R. pusillus* relative to the corresponding species, whereas negative values indicate lower expression in *R. pusillus*.

To investigate the distinctive functional features of the immune system in *R. pusillus* compared with other mammals associated with zoonotic infections, we performed a multi-species comparative transcriptomic analysis. This analysis integrated newly generated sequencing data with publicly available datasets to construct splenic expression matrices for ten mammalian species (**Supplementary Table 6**). For each species, the top 1,000 most highly expressed genes were selected and subjected to GO enrichment analysis to identify the most prominently represented functional patterns in basal expression across species. *R. pusillus* exhibited the strongest enrichment in multiple immune-related categories, including innate immune response (GO:0045087), positive regulation of immune response (GO:0050778), regulation of innate immune response (GO:0045088), and activation of immune response (GO:0002253) (**Figure 1A**). In addition, genes involved in innate immune regulation and antiviral immune responses were markedly active in *R. pusillus*, whereas genes associated with the regulation of inflammatory response were relatively attenuated compared with humans, mice, and other bat species included in this analysis (**Figure 1B**).

To directly investigate transcriptional differences between *R. pusillus* and other species, differential expression analyses of spleen expression matrices were performed between *R. pusillus* and each of the nine other species. GO enrichment analysis of upregulated genes revealed that, compared with *R. ferrumequinum*, *R. pusillus* exhibited a markedly elevated basal immune response (**Supplementary Figure 3A**). Although its overall immunological advantage relative to other species was less pronounced, multiple IRGs associated with innate immune response terms were consistently upregulated across all comparisons (**Supplementary Figures 3B, C**). To further characterize the basal antiviral immune features of *R. pusillus*, IRGs reported in multiple bat comparative immunology studies were compiled, encompassing genes involved in the classical complement systems as well as interferon-mediated antiviral innate immunity pathways (**Figure 1C-E**). The results showed that most of these immune genes were differentially expressed between *R. pusillus* and other species, with 90% being upregulated in *R. pusillus*, indicating a consistently elevated basal antiviral gene expression in *R. pusillus* relative to each compared species across all pairwise analyses (**Figure 1F**).

### Transcriptomic profiling of *R. pusillus* by WGCNA

**Figure 2:**
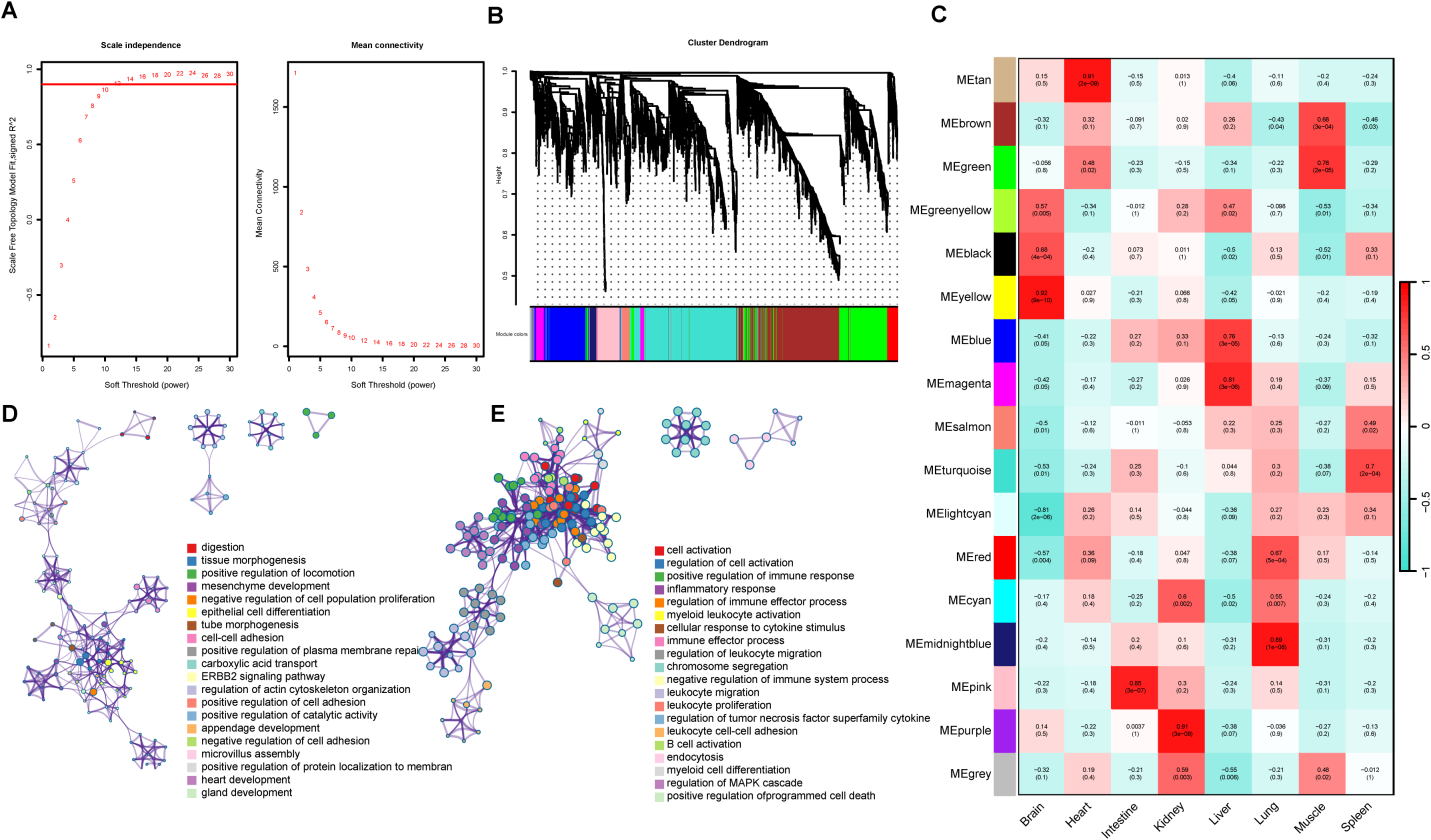
WGCNA analysis of the multi-tissue transcriptomic atlas of *R. pusillus.* **(A)** Analyses of scale-free fit index and mean connectivity for various soft-thresholding powers (β). **(B)** Cluster dendrogram of modules identified using the Dynamic Tree Cut algorithm. Functional modules are represented in different colors, each major branch represents a color-coded module that contains a group of highly connected genes. **(C)** Heatmap between 17 modules and 8 tissues. Boxes display Pearson correlation coefficients and their associated P values. Red indicates that the given tissue has a strong positive correlation relative to all other tissues. Blue indicates that the given tissue has a strong negative correlation relative to other tissue. The X-axis represents tissue types, and the Y-axis represents modules of different colors. **(DE)** Enrichment_GO_ColorByCluster analyses of the intestine and spleen tissue-speci2fic modules by Metascape.

As a potential model for studying virus-host interactions and host viral tolerance, characterizing tissue-specific transcriptional profiles in *R. pusillus* is essential for understanding its biological features and specialized immune functions. To dissect the molecular basis underlying functional differentiation among tissues, WGCNA was performed on transcript atlas derived from multiple tissues of *R. pusillus*. As shown in **Figure 2A**, power value 12, which was the lowest power for the scale‐free topology fit index on 0.9, was selected to produce a hierarchical clustering tree. Then WGCNA was used to assign genes with similar expression patterns into one module, and 17 gene modules were obtained (**Figure 2B**). A dendrogram and heatmap were used to quantify module similarity by correlation. Tissue-specific modules were identified based on the correlation between module and tissue type (**Figure 2C**).

Functional enrichment analysis revealed the diverse functions of tissue-specific modules. The intestinal module was enriched in processes associated with tissue morphogenesis, epithelial cell differentiation, microvillus assembly, cell adhesion, and plasma membrane maintenance, reflecting the intestine’s specialized roles in structural development, barrier integrity, and cellular homeostasis (**Figure 2D**). In contrast, the spleen-specific module showed significant enrichment for immune-related processes, including positive and negative regulation of immune system process, leukocyte migration and proliferation, immune effector process, and inflammatory responses, together with cytokine-responsive pathways such as regulation of the tumor necrosis factor superfamily and the MAPK cascade (**Figure 2E**), highlighting its central role in coordinating complex immune regulatory networks.

In addition to the spleen, other tissue-specific modules also displayed enrichment patterns that reflect both physiological functions and context-dependent immune activities. Notably, the liver-associated module showed enrichment in immune-related processes, including inflammatory response, regulation of complement activation, cellular response to cytokine stimulus, and positive regulation of phagocytosis, suggesting its involvement in systemic immune modulation and host defense. Other tissues, such as kidney and muscle, also exhibited enrichment of immune-related terms (e.g., regulation of leukocyte activation and response to wounding), indicating that immune-related processes are distributed across multiple organs. Together, these findings suggest that immune regulation in *R. pusillus* involves coordinated contributions from multiple tissues, with the spleen acting as a central immune organ and other tissues providing complementary roles in local immune responses and immune signaling, thereby forming an integrated multi-tissue immune network that may support viral tolerance (**Supplementary Figure 4**).

### Differential expression analysis between *R. pusillus* and *R. ferrumequinum*

*R. pusillus* and *R. ferrumequinum* are widely distributed horseshoe bats that serve as important natural reservoir hosts for diverse coronaviruses. However, compared with *R. ferrumequinum*, the unique genetic differentiation of *R. pusillus* and its role as a “recombination incubator” for diverse sarbecoviruses suggest that it may have evolved species-specific functional adaptations among horseshoe bats. To explore this hypothesis, we performed differential expression analysis across eight tissues from two species (**Supplementary Table 7**).

**Figure 3:**
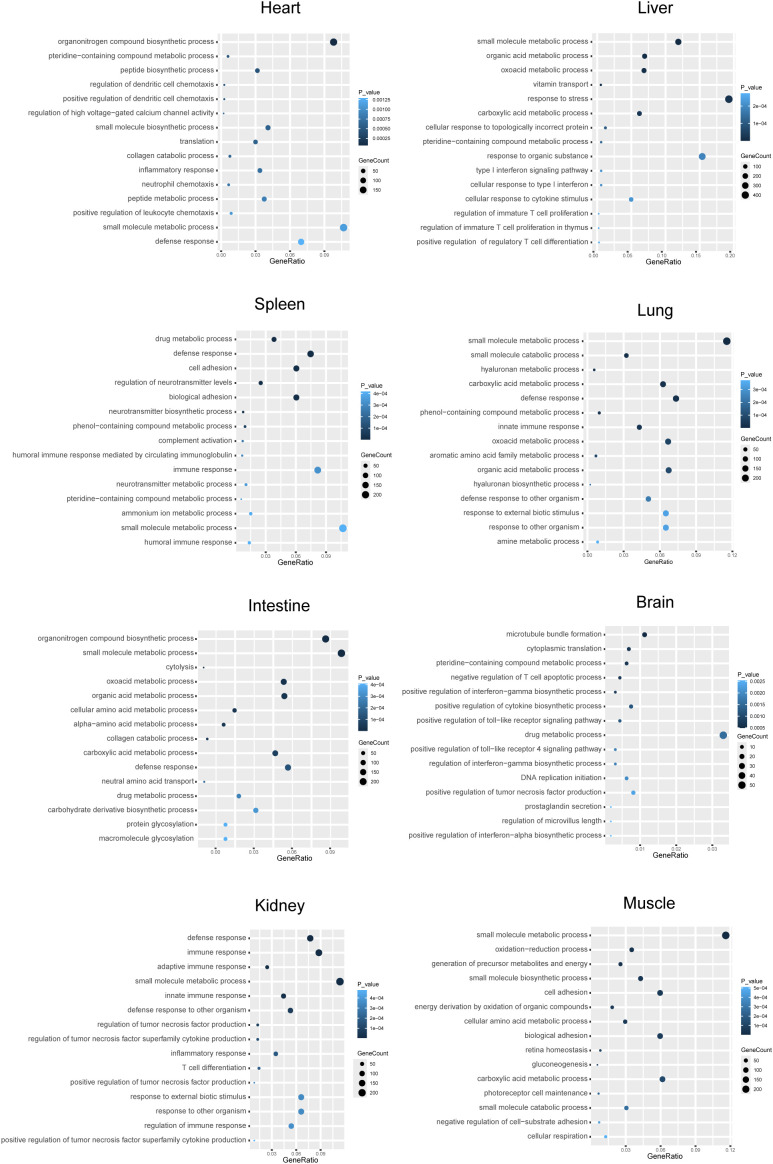
GO enrichment analysis of differentially expressed genes **(DEGs)** between *R. pusillus* and *R. ferrumequinum.* The bubble chart displays the top 15 enriched GO terms. The x-axis represents the GeneRatio, and the y-axis represents GO terms. Bubble size indicates the number of genes associated with each term, while color intensity indicates the significance level **(p-value)** of GO term enrichment.

We then performed GO enrichment analysis on DEGs from each tissue to explore their potential distinct physiological functions. The 15 most significantly enriched GO terms were visualized in a bubble chart (**Figure 3**). Results revealed pronounced differences between *R. pusillus* and *R. ferrumequinum* across multiple tissues. In the liver, intestine, lung, and heart, DEGs were predominantly associated with metabolic processes, including small-molecule metabolism, organic and carboxylic acid metabolism, and oxoacid metabolism, reflecting distinct metabolic regulatory patterns between the two species. The kidney, liver, and lung tissues were all enriched for pathways related to adaptive and innate immune responses, further highlighting a coordinated contribution of multiple organs to systemic immunity. The spleen displayed diverse immune features, encompassing innate immunity (e.g., defense response, complement activation), humoral immunity, and immune cell adhesion and recruitment processes. Additionally, several of the most significantly differentially expressed genes in the spleen, including upregulated *GPBAR1*, *MSS51*, *CDH24*, and *LSR*, and downregulated *RPS12*, *RPL24*, *HNRNPR*, *ZRANB2*, *SFRP1*, and *FKBP1A*, further highlight molecular differences that may underlie tissue-specific immune and physiological specialization. Taken together, these findings suggest that the spleen of *R. pusillus* exhibits multilayered immune specialization, which likely operates in coordination with metabolic and immune processes in other organs to support systemic immune defense.

### Identification of innate immune hub genes in *R. pusillus*

**Figure 4:**
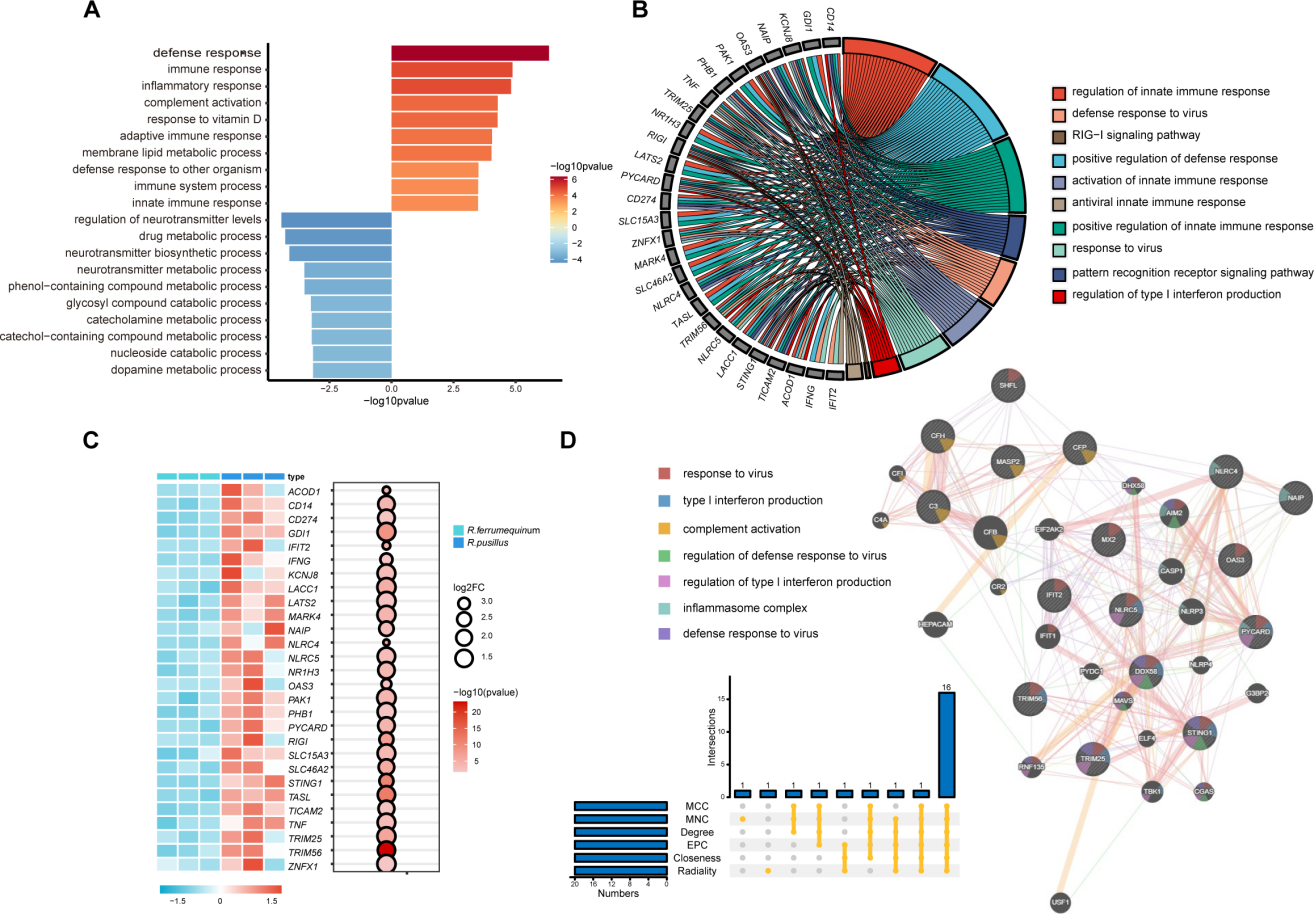
Identification of molecular mechanisms underlying antiviral innate immunity in the *R. pusillus* spleen. **(A)** Functional annotation of up- and down-regulated DEGs in the spleen of *R. pusillus*. Red bars indicate GO terms enriched in upregulated genes, whereas blue bars represent GO terms enriched in downregulated genes. Color intensity reflects the significance level of enrichment (p-value). **(B)** Chord diagram illustrates the correlations between antiviral innate immune-related genes and their associated biological process **(BP)** terms. **(C)** Differential expression patterns of antiviral innate immune-related genes in spleen tissues of *R. pusillus* and *R. ferrumequinum*. **(D)** Protein-protein interaction **(PPI)** network of genes associated with the innate immune response. Hub genes are identified from the entire PPI network using the cytoHubba plugin in Cytoscape based on the MCC, Degree, EPC, Closeness, Radiality, and MNC algorithms. The GeneMANIA database is further employed to predict genes or proteins that potentially interact with the identified hub genes.

To further investigate the mechanisms underlying viral infection tolerance in *R. pusillus*, functional enrichment analysis was performed on DEGs identified in spleen samples from the two horseshoe bat species. The top 10 GO terms for up- and downregulated genes are shown in **Figure 4A**. Genes upregulated in *R. pusillus* were predominantly enriched in immune response-related terms, indicating transcriptionally enhanced immune responses in *R. pusillus*. In contrast, upregulated genes in *R. ferrumequinum* were primarily linked to metabolic processes, indicating enrichment of metabolic functions in this species.

Given the central role of innate immunity in early antiviral defense, we further examined innate immune responses in *R. pusillus*. Innate immune-related pathways were significantly enriched in the spleen of *R. pusillus*, with multiple immune genes concurrently involved in antiviral processes (**Figure 4B, C**). Protein-protein interaction analysis based on the “innate immune response” GO term identified several immune-related hub genes, primarily associated with viral recognition, interferon signaling, and downstream antiviral effector mechanisms (**Figure 4D**, **Supplementary Table 8**). Notably, *DDX58* (*RIG-I*) and *TRIM25* are both core components of the canonical *RIG-I* mediated viral RNA recognition pathway, in which viral RNA sensing and *TRIM25*-dependent ubiquitination activate downstream signaling to initiate type I interferon responses. *STING1*, as a key adaptor molecule in the cGAS-STING signaling axis, may further amplify immune signaling following viral nucleic acid recognition. Downstream antiviral effector genes, including *MX2*, *OAS3*, and *IFIT2*, were significantly enriched and directly participate in suppressing viral replication, inhibiting viral protein translation, or promoting viral RNA degradation, collectively forming a multilayered intracellular antiviral defense system.

### LncRNA regulation of immune signatures in *R. pusillus*

**Figure 5:**
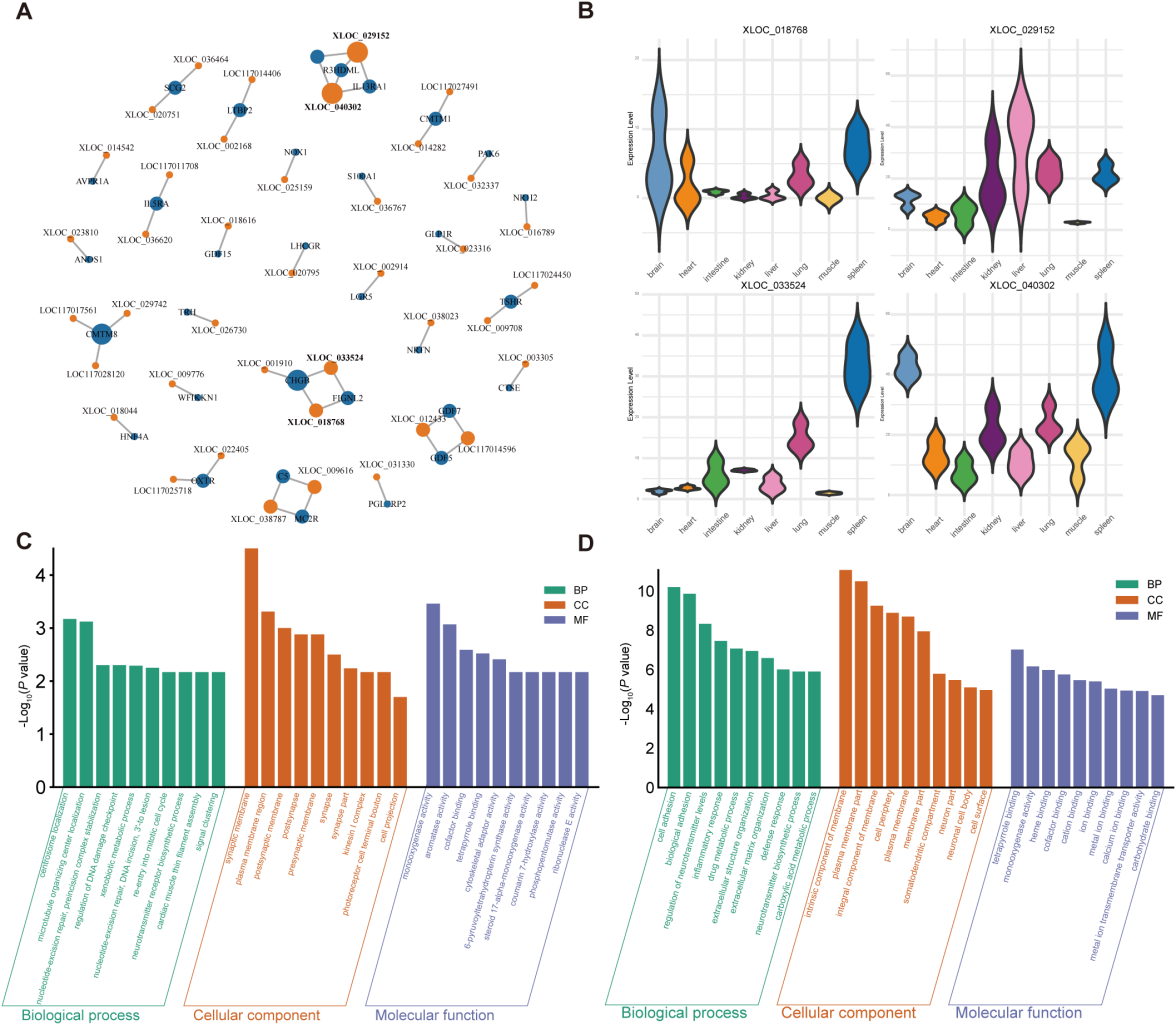
Prediction of lncRNAs regulating the immune response in *R. pusillus.* **(A)** Regulatory network of immune-related genes and lncRNAs based on expression pattern consistency. The orange node denotes lncRNA and the blue node represents immune-related gene, and genes with more connections indicate higher centrality. **(B)** Expression levels of hub lncRNA transcripts across tissues is visualized using violin plots. **(CD)** Distribution of GO terms for cis-regulated and trans-regulated targets in Biological Process, Cellular Component, and Molecular Function.

To elucidate the regulatory mechanisms underlying the immune-related transcriptional signatures observed in *R. pusillus*, we predicted lncRNAs across multiple tissues from the two horseshoe bat species. By integrating expression matrices of IRGs and lncRNAs, we constructed lncRNA-IRG coexpression network to identify candidate lncRNAs potentially involved in immune regulation. In this network, XLOC_018768 and XLOC_033524 were coexpressed with *CHGB* and *FIGNL2*, whereas XLOC_029152 and XLOC_040302 were associated with *R3HDML* and *IL13RA1*(**Figure 5A**). These hub lncRNAs exhibited pronounced tissue-specific expression patterns. Although all identified hub lncRNAs were consistently expressed in the spleen, XLOC_018768 showed relatively high expression levels in brain and heart, whereas XLOC_033524 was scarcely expressed in these tissues (**Figure 5B**). The function of this lncRNA-mRNA coexpression requires further research.

As lncRNAs do not encode proteins, their regulatory functions are primarily mediated through cis-acting elements and trans-acting mechanisms affecting protein-coding genes. We performed both cis- and trans-target analyses for differentially expressed lncRNAs, and inferred their potential functions through functional enrichment analysis of the predicted target genes (**Figure 5C, D**). Target genes of differentially expressed cis-acting lncRNAs were mainly enriched in the nucleotide-excision repair and regulation of DNA damage checkpoint, indicating a regulatory role for lncRNAs in DNA damage responses. Target genes of trans-acting lncRNAs were predominantly enriched in inflammatory response, defense mechanisms, cell adhesion, and extracellular matrix organization, indicating potential regulation of immune activation while preserving tissue integrity. Together, these lncRNAs likely contribute to the anti-inflammatory state and tolerance to tissue damage of *R. pusillus*.

## Discussion

Bats are widely recognized as natural hosts of many zoonotic viruses with pandemic potential, yet the molecular basis underlying their remarkable capacity for viral tolerance remains poorly understood. Notably, when challenged by these viruses, bats often exhibit no or only minimal signs of disease, even in the presence of high viral loads in sera or tissues. These observations have prompted growing interest and efforts to characterize the bat immune system, primarily through genomic (31, 32) and transcriptomic analyses (33–35). In this study, we performed a comprehensive multi-tissue and cross-species comparative transcriptomic analysis to characterize the immune features of *R. pusillus*. By integrating comparative transcriptomics, tissue-level co-expression network analyses, and immune-associated lncRNA-mRNA regulatory network inference, we identify a coordinated transcriptional regulatory architecture that supports innate immune responses while restraining excessive inflammatory activity and maintaining tissue homeostasis. These findings suggest that viral tolerance represents a systems-level feature of immune regulation, reflecting evolutionary adaptations that enable bats to tolerate persistent viral exposure.

Previous studies have highlighted distinctive features of antiviral immunity in bats, particularly the elevated basal expression of genes involved in complement activation and innate immune signaling. For example, organoid studies in *Rousettus aegyptiacus* reported constitutively high expression of genes associated with both the classical and alternative complement pathways (36). Consistent with these findings, our analysis showed that several complement-related genes, including *C2*, *C3*, *C6*, *CFB*, and *CFH*, exhibit higher basal expression levels in bats compared with other mammals, suggesting a potential role for complement activation in antiviral defense. In addition, previous studies have demonstrated widespread expression of *IRF7* and elevated basal levels of antiviral sensing and response genes in bats (37, 38). Similarly, we observed sustained high expression of multiple antiviral genes, including *TLR3*, *TLR7*, *MX1*, *MX2*, *IFIT3*, and *OAS3*, in *R. pusillus*, supporting the presence of a pre-activated antiviral state. Notably, while elevated basal expression of multiple interferon types has been reported in bat organoid systems (39), our data showed that only *IFNG* exhibited significantly higher basal expression in *R. pusillus*, whereas other interferons were below the detection threshold. This difference may reflect variation in experimental systems or the inherently low and tightly regulated expression of interferons under basal conditions. We observed significant enrichment of interferon signaling pathways and elevated expression of interferon-stimulated genes (ISGs) in *R. pusillus*, in agreement with previous studies (40, 41), supporting a model in which constitutive antiviral gene expression contributes to viral tolerance in bats.

Tissue-specific co-expression network analysis provides a global view of the functional landscape, suggesting that immune functions in *R. pusillus* are coordinated with multiple biological systems. The spleen-specific module indicates that immune activation and regulatory processes are integrated within a shared transcriptional framework, potentially enabling a balanced immune state in which antiviral responses are maintained while excessive activation is restrained. Beyond the spleen, other tissues exhibit enrichment patterns consistent with their physiological roles, including energy metabolism and mitochondrial processes in muscle, small-molecule metabolism and inflammatory regulation in the liver, developmental and signaling pathways in the lung, and homeostatic regulation and stress responses in the kidney. Heart and brain associated modules were enriched for muscle contraction and neuronal signaling, respectively, further supporting tissue-specific functional specialization. Together, these findings indicate that immune regulation is not confined to a single organ but operates within a coordinated multi-tissue framework, where organ-specific functions and systemic signaling are integrated. Such organization may enhance immune robustness under persistent viral exposure. Although the underlying mechanisms remain to be elucidated, this study provides a foundation for understanding the multi-tissue functional architecture in this species.

Differential gene expression analysis provides insight into molecular mechanisms that may contribute to viral tolerance in *R. pusillus*. Notably, this species uniquely harbors multiple sarbecovirus lineages, a pattern not observed in other closely related horseshoe bats. To investigate transcriptional features associated with this distinction, we performed a comparative analysis with *R. ferrumequinum*, a closely related species with a well-annotated genome and comparatively limited sarbecovirus diversity (15). As a relatively large-bodied horseshoe bat, *R. ferrumequinum* is widely distributed and harbors multiple coronaviruses. however, it appears to have played a significant role only in the evolutionary history of SARSr-CoVs, with no evidence of carrying the other two major sarbecovirus lineages (42, 43). Despite the close evolutionary relationship, these two species differ markedly in their viral repertoires, allowing us to focus on transcriptional features in *R. pusillus* that may underlie its unique capacity to tolerate multiple sarbecoviruses. Across multiple tissues, *R. pusillus* exhibited distinct transcriptional profiles characterized by differences in immune signaling, antiviral responses, and metabolic regulation. In the spleen, genes upregulated in *R. pusillus* were predominantly associated with innate and antiviral immune responses, whereas genes more highly expressed in *R. ferrumequinum* were enriched for metabolic processes. This contrast shows *R. pusillus* may maintain a transcriptional state that is more readily poised for activation of antiviral innate immune responses, without a concomitant increase in metabolic activation, potentially mitigating the energetic demands associated with sustained immune activity. Overall, these findings point to coordinated, multi-system transcriptional adaptations in *R. pusillus* that may confer functional advantages in balancing antiviral defense and physiological homeostasis.

Focusing on the spleen, we identified a set of innate immune-related hub genes that form a densely connected protein-protein interaction network in *R. pusillus*, highlighting species-specific antiviral transcriptional programs. Cytosolic pattern recognition receptor *RIG-I (DDX58)* was highly expressed in *R. pusillus*, initiating downstream antiviral signaling, while the stimulator of interferon genes (*STING*), a key adaptor in DNA sensing pathways (44), was also upregulated. High *STING* expression may balance antiviral activity with limited inflammatory signaling, as STING-mediated type I interferon induction is dampened in bat cells due to structural adaptations (9). *NLRP3* has been functionally characterized in bats, whereas *NLRC5* has only been detected at the transcript level in *Pteropus alecto* (34). *NLRC5* may be involved in cytokine responses and antiviral immunity, potentially through inhibition of NF-kappa-B activation and negative regulation of type I interferon signaling, analogous to roles played by NLRP3 in immune regulation (7). In addition, the differential expression profiles of the spleen also contained many important antiviral proteins encoded by ISGs, a class of antiviral cellular factors induced by type I interferons (IFN) that include *MX2, OAS3, IFIT2, TRIM25*, and *TRIM56*. Among these, *TRIM25* stimulates innate immune signaling by ubiquitinating and activating *RIG-I*, playing critical roles in both antiviral immunity and cancer defense (45–47). Among the top upregulated genes, *GPBAR1*, a bile acid receptor expressed in innate immune cells that promotes a tolerogenic state and suppresses NF-kappa-B signaling to limit inflammatory responses (48, 49), and *MSS51*, involved in mitochondrial function and energy metabolism, together with CDH24, a cadherin mediating cell-cell adhesion, may contribute to tissue homeostasis and immune regulation. In contrast, downregulated genes, including ribosomal components *(RPS12*, *RPL24*) and RNA-processing factors (*HNRNPR*, *ZRANB2*), indicate a reduced translational and biosynthetic state. The downregulation of *SFRP1 (*50), a secreted Wnt antagonist that can activate pro-inflammatory pathways in innate immune cells, and *FKBP1A*, involved in cellular signaling, may further modulate immune activity.

Beyond protein-coding genes, this study highlights a substantial contribution of lncRNAs to the immune regulatory landscape of *R. pusillus*. Immune-associated lncRNAs exhibited pronounced tissue specificity and formed extensive co-expression networks with immune genes. Functional inference based on predicted cis- and trans-regulated targets suggests that these lncRNAs participate in diverse biological processes. Some hub lncRNAs were associated with *IL13RA1*, a subunit of the interleukin 13 receptor that forms receptor complexes with *IL4* receptor alpha and mediates signaling through *JAK1*, *STAT3*, and *STAT6* in response to *IL13* and *IL4*, potentially influencing immune cell activation and inflammatory responses. Others were linked to *CHGB*, a tyrosine-sulfated secretory protein abundant in endocrine cells and neurons that may serve as a precursor for regulatory peptides, suggesting a role in modulating cellular secretion and maintaining tissue homeostasis. The enrichment of cis-acting lncRNA targets in DNA damage repair and checkpoint regulation points to enhanced control of genome stability, likely reflecting adaptation to oxidative and metabolic stress. In parallel, trans-acting lncRNAs were associated with inflammatory and defense-related pathways, indicating a role in fine-tuning immune activation while preserving tissue integrity. This coordinated regulatory landscape highlights a unique balance between viral tolerance and physiological homeostasis.

Several limitations of this study should be acknowledged. This study included only two bat species, with three individuals per species, and variation in capture locations may introduce inter-individual variability. The limited sample size reflects ecological constraints of bat species, strict wildlife sampling regulations in China, and stringent quality and inclusion criteria applied to field-collected samples. Therefore, the results should be interpreted as preliminary conclusions, and the characteristics of the entire population require further investigation. The study is based on transcriptomic data from naturally sampled, apparently healthy individuals, and thus do not capture dynamic immune responses during acute infection or under experimental viral challenge. In addition, functional validation of candidate hub genes and immune-associated lncRNAs will be necessary to directly establish their roles in antiviral defense and immune regulation. Future studies integrating single-cell transcriptomics, epigenetic profiling, and functional assays will further refine our understanding of cell-type-specific immune strategies in *R. pusillus*.

## Data Availability

The datasets presented in this study can be found in online repositories. The transcriptome data of the two horseshoe bat species have been deposited in the National Genomics Data Center (NGDC) under accession number PRJCA054585. Some datasets presented in this article are not readily available because they are associated with an ongoing unpublished study and are currently under further analysis. The genome data of R. pusillus have been submitted to NGDC under accession number PRJCA045276. Requests to access these datasets should be directed to the corresponding authors.

## Supplementary material

Supplementary Figure 1 Virome profiling workflow.

Supplementary Figure 2 Field sampling map of bats in this study. Colors represent different bat species, and pie chart size represents sample size.

Supplementary Figure 3 Differential expression analysis of spleen tissues between *R. pusillus* and nine other mammalian species. **(A)** GO enrichment analysis of genes upregulated in *R. pusillus* relative to *R. ferrumequinum*. **(B)** GO enrichment analysis of genes upregulated in *R. pusillus* relative to other mammalian species. **(C)** Innate immune GO term genes upregulated in *R. pusillus* across all comparison groups.

Supplementary Figure 4 Enrichment analyses of the remaining six tissue-specific modules, performed using Metascape and colored by cluster.
