# Supplementary figures and images for: Comparative transcriptome analysis unveils unique antiviral immune signatures of Rhinolophus pusillus
Source: Front Immunol. 2026 May 20;17:1797102. doi: 10.3389/fimmu.2026.1797102 (PMC13229797; doi:10.3389/fimmu.2026.1797102)

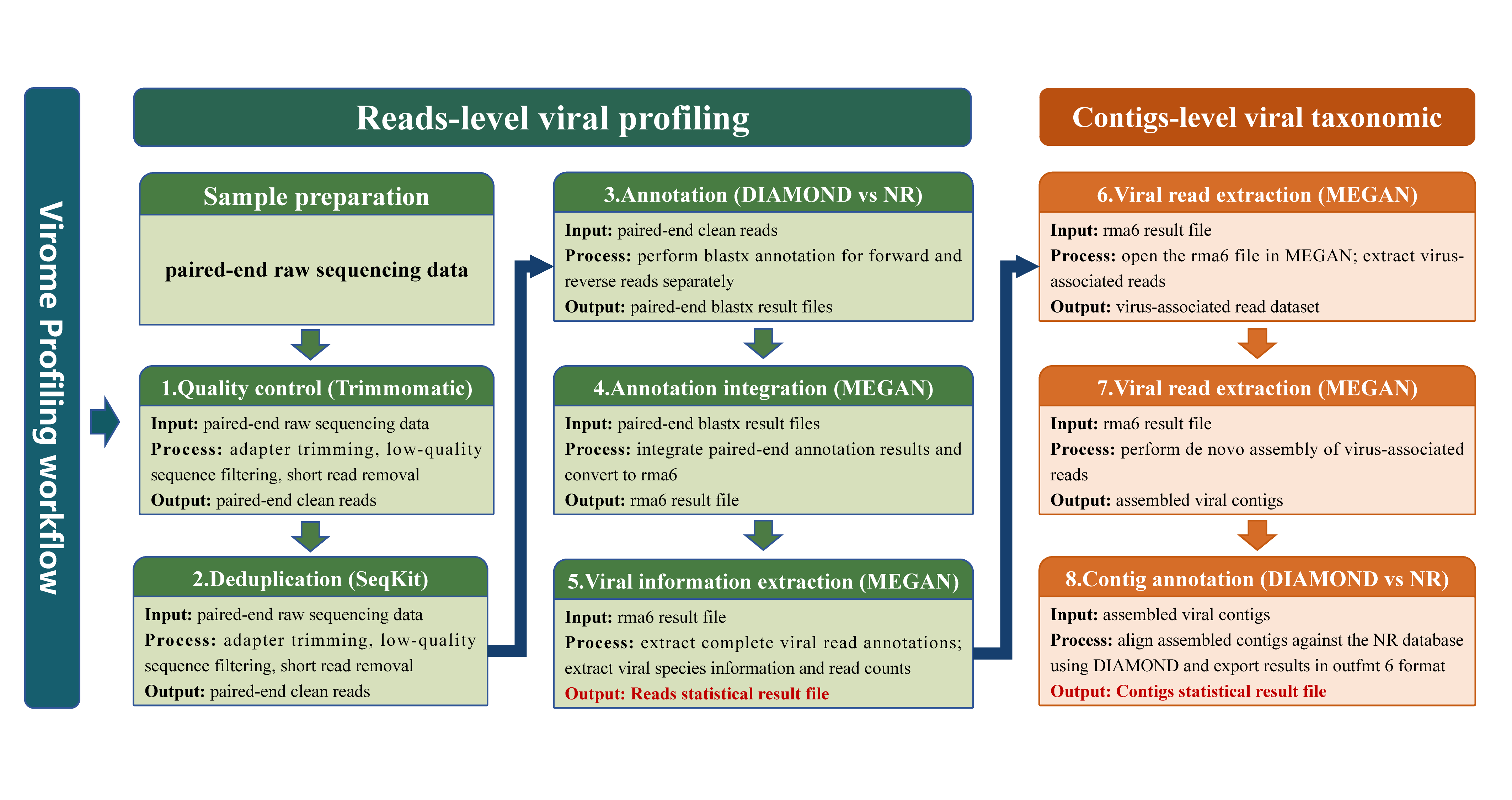

Supplement: Supplementary Figure 1 — Virome profiling workflow. [file Image1.tif]

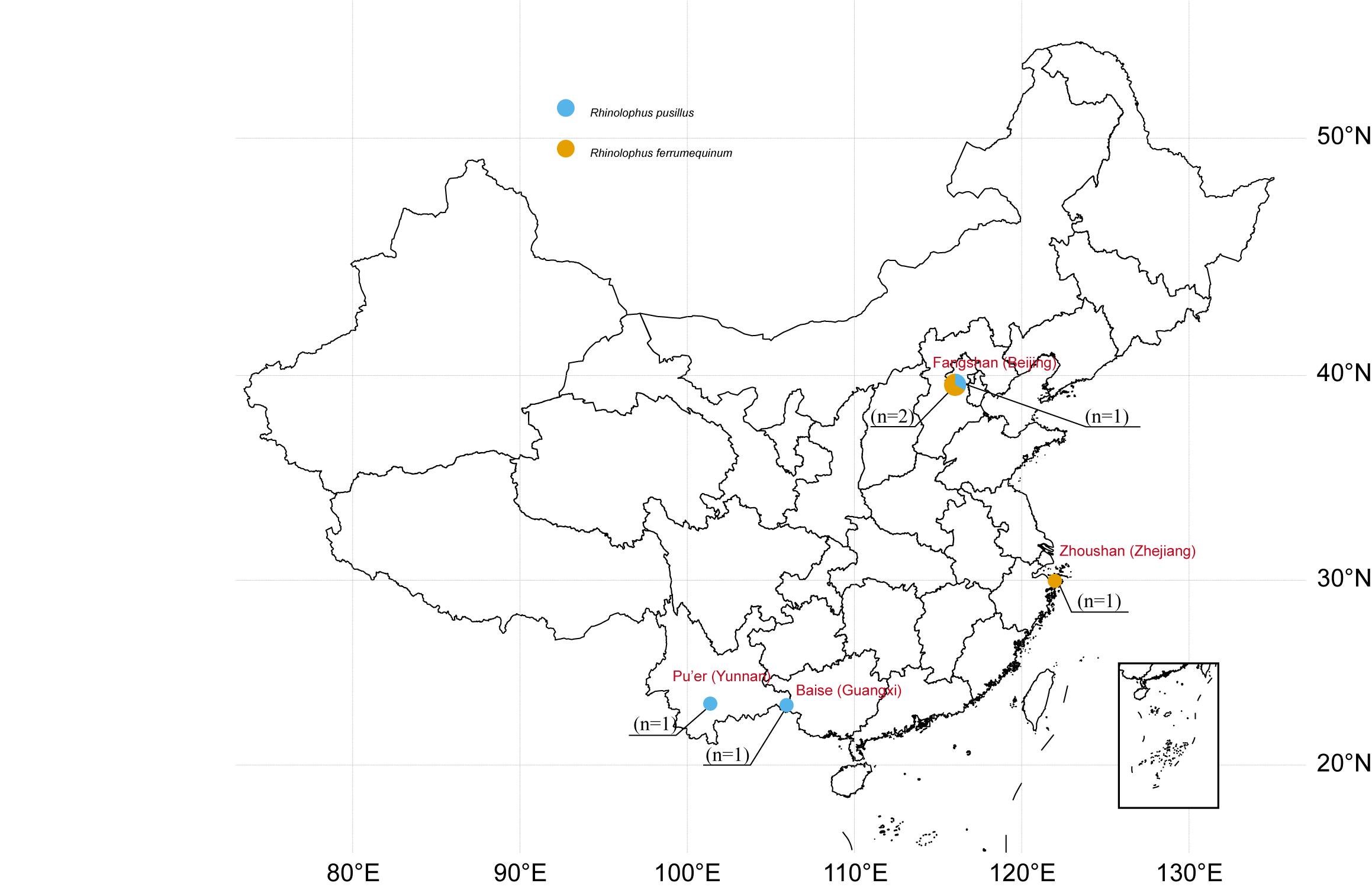

Supplement: Supplementary Figure 2 — Field sampling map of bats in this study. Colors represent different bat species, and pie chart size represents sample size. [file Image2.tif]

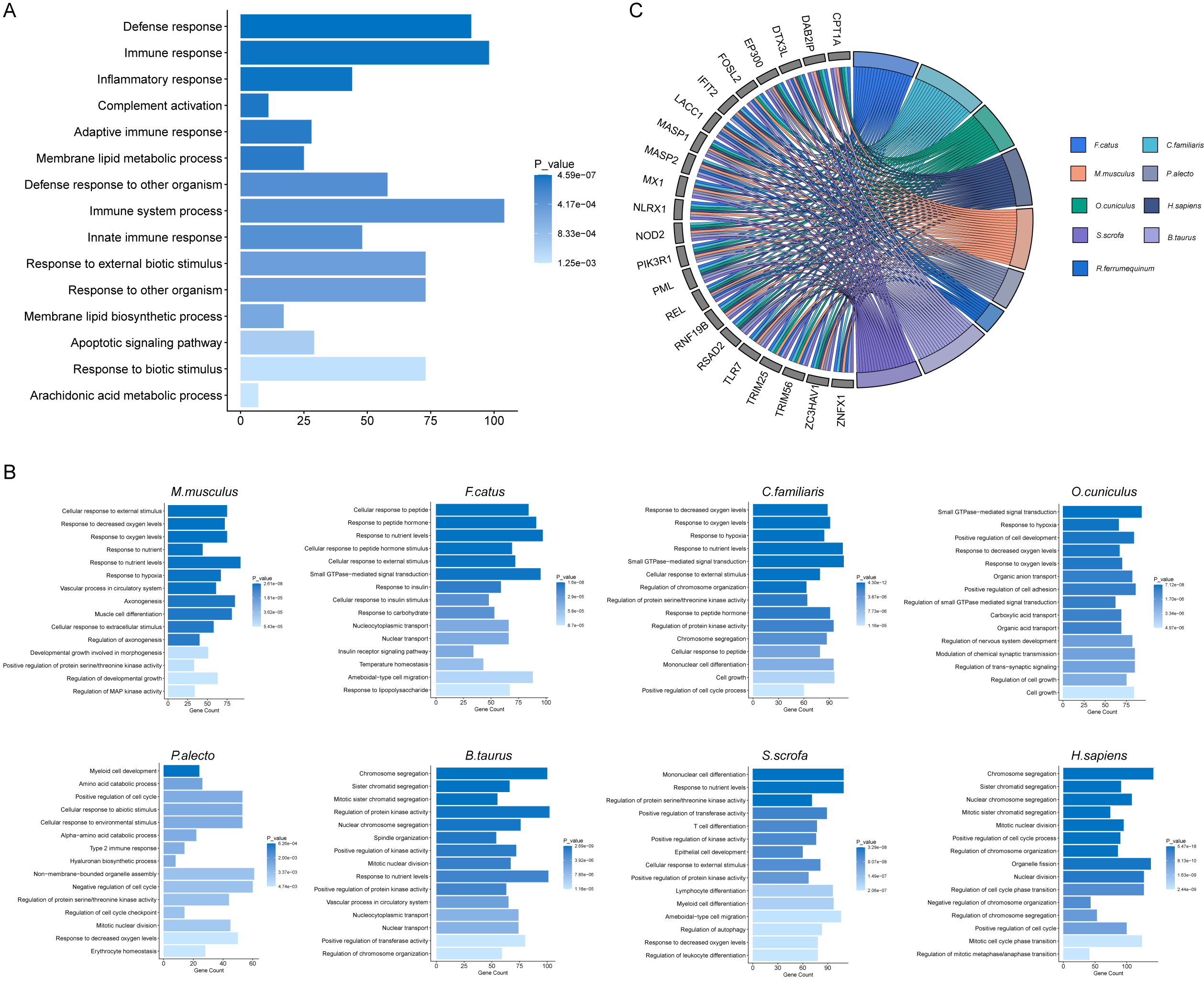

Supplement: Supplementary Figure 3 — Differential expression analysis of spleen tissues between R. pusillus and nine other mammalian species. (A) GO enrichment analysis of genes upregulated in R. pusillus relative to R. ferrumequinum. (B) GO enrichment analysis of genes upregulated in R. pusillus relative to other mammalian species. (C) Innate immune GO term genes upregulated in R. pusillus across all comparison groups. [file Image3.tif]

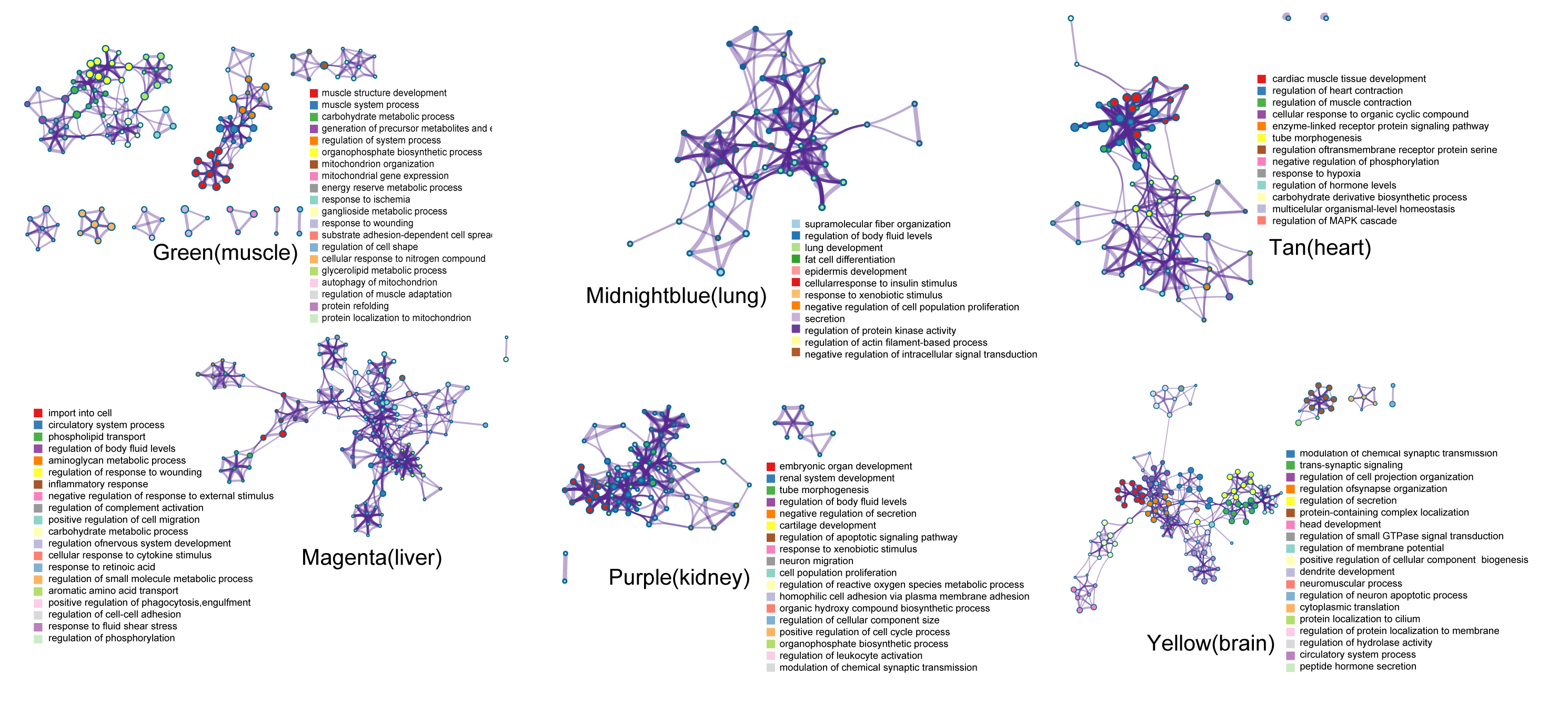

Supplement: Supplementary Figure 4 — Enrichment analyses of the remaining six tissue-specific modules, performed using Metascape and colored by cluster. [file Image4.tif]
